# Exploring the Assembly of Resorc[4]arenes for the Construction of Supramolecular Nano-Aggregates

**DOI:** 10.3390/ijms222111785

**Published:** 2021-10-29

**Authors:** Fabio Buonsenso, Francesca Ghirga, Isabella Romeo, Gabriella Siani, Serena Pilato, Deborah Quaglio, Marco Pierini, Bruno Botta, Andrea Calcaterra

**Affiliations:** 1 Department of Chemistry and Technology of Drugs, “Department of Excellence 2018−2022”, Sapienza University of Rome, P. le Aldo Moro 5, 00185 Rome, Italy; fabio.buonsenso@uniroma1.it (F.B.); francesca.ghirga@uniroma1.it (F.G.); isabella.romeo@uniroma1.it (I.R.); bruno.botta@uniroma1.it (B.B.); andrea.calcaterra@uniroma1.it (A.C.); 2 Center for Life Nano and Neuroscience, Italian Institute of Technology, Viale Regina Elena 291, 00161 Rome, Italy; 3 Department of Pharmacy, University of Chieti “G. D’Annunzio”, Via dei Vestini 31, 66013 Chieti, Italy; gabriella.siani@unich.it (G.S.); serena.pilato@unich.it (S.P.)

**Keywords:** resorc[4]arenes, supramolecular assembly, solubilizing agents, Glabrescione B, UV-vis spectroscopy

## Abstract

Many biologically active compounds feature low solubility in aqueous media and, thus, poor bioavailability. The formation of the host-guest complex by using calixarene-based macrocycles (i.e., resorcinol-derived cyclic oligomers) with a good solubility profile can improve solubilization of hydrophobic drugs. Herein, we explore the ability of resorc[4]arenes to self-assemble in polar solutions, to form supramolecular aggregates, and to promote water-solubility of an isoflavone endowed with anti-cancer activity, namely Glabrescione B (GlaB). Accordingly, we synthesized several architectures featuring a different pattern of substitution on the upper rim including functional groups able to undergo acid dissociation (i.e., carboxyl and hydroxyl groups). The aggregation phenomenon of the amphiphilic resorc[4]arenes has been investigated in a THF/water solution by UV–visible spectroscopy, at different pH values. Based on their ionization properties, we demonstrated that the supramolecular assembly of resorc[4]arene-based systems can be modulated at given pH values, and thus promoting the solubility of GlaB.

## 1. Introduction

**Figure 1:**
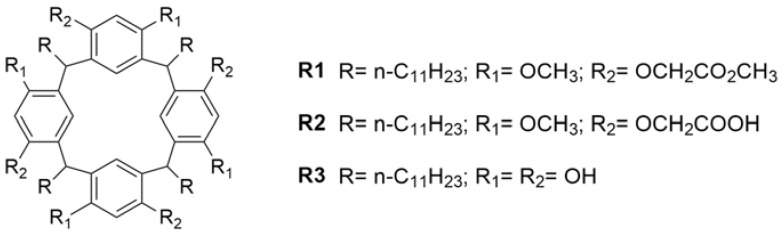
Chemical structure of amphiphilic resorc[4]arenes **R1**, **R2,** and **R3**.

A wide range of biologically active compounds suffers from poor aqueous solubility impairing their bioavailability and, as a result, their preclinical and clinical development. The self-assembly process of well-defined structures from various chemical building blocks have found exponential growth in the development of drug delivery and bio-nanotechnology systems [1]. In particular, these assemblies give the possibility to encapsulate pharmaceutically active compounds in their core (or at their surface) and to cargo them to the therapeutic targets [2]. Self-assembly can include different levels of complexity: it can be as simple as the dimerization of two small building blocks driven by hydrogen bonding or more complicated as a cell membrane [1], a remarkable supramolecular architecture created by a bilayer of phospholipids embedded with functional proteins. In addition to a vast series of natural amphiphilic structures, several ‘‘engineered’’ synthetic architectures have been designed as solubilizing agents, using a macrocyclic core such as cyclodextrins and crown-ethers [2,3,4,5,6,7,8,9,10,11,12,13]. Among the large pool of macrocycles available, calixarene-based macrocycles are one of the most ubiquitous host molecules in supramolecular chemistry. These macrocycles are cyclic oligomers characterized by a unique three-dimensional surface, featuring several phenolic units bound with methylene bridges, which can form large hydrophobic cavities. The high versatility of their chemical structure, which can be variously functionalized at both the upper and lower rims, allows the combination of hydrophilic and hydrophobic groups favoring the amphiphilic behavior of macrocycles and the formation of self-associates with a varying morphology [14]. Supramolecular complexation techniques using calixarene-based macrocycles as hosts may improve not only the solubility but also the stability of the guest molecules (including drugs), several examples of calixarene-guest aggregates have been reported [15,16,17]. In order to overcome the low aqueous solubility of these macrocycles, the upper or lower rim can be easily functionalized with water soluble groups containing positive or negative charges [18,19,20,21,22,23,24]. Among them, sulfonated calixarenes have dominated drug solubility studies [16]. Within the calixarene family, resorcinol-derived cyclic oligomers, namely resorc[4]arenes, endowed with cavities of molecular dimension, behave as an efficient artificial receptor [14,25,26]. The synthetic modularity and different complexation properties of the resorcarene-based macrocycles make them a promising building block for the design and synthesis of more sophisticated supramolecular architectures [27,28,29,30,31]. Efficient solubilizing agents were designed and developed by using several resorc[4]arenes [15,32,33]. Recently, Morozonova et al. reported that aggregates of amphiphilic calix-resorcinarenes, featuring amidoamino and dimethylamino peripheral groups on the upper rim and different aliphatic groups (pentyl, octyl, and undecyl) on the lower rim, behave as effective solubilizing agents of hydrophobic drugs containing a carboxyl group (e.g., naproxen, ibuprofen, and ursodeoxycholic acid) [34]. The driving force of the association process is the ionization of organic acids and the peripheral nitrogen atoms of the macrocycles with the subsequent inclusion of hydrophobic acids into the macrocycle self-associates. Intriguingly, the solubilization of carboxylic acids in more than an equimolar ratio leads to the co-assembly of the macrocycle polydisperse associates into supramolecular monodisperse nanoparticles with the diameter of about 100 nm [34]. However, to date, a limited use of resorcarene derivatives as an amphiphilic host to complex poorly water-soluble drugs has been made with the purpose of enhancing their water-solubility and bioavailability. In a previous study, we demonstrated that physical descriptors, namely the aggregation polarity index (**API**), the cavitation Gibbs free-energy change (ΔΔGcav), and the decrease of the molecular surface (A) after the exposure of solute to the solvent upon aggregation (%ΔA), were able to monitor the propensity of a double-spanned resorc[4]arene derivative (**BSK**), featuring a basket-like structure, to self-assembly in tetrahydrofuran (THF)/water solutions [29]. Based on these findings, with the aim to investigate the self-aggregation propensity of resorc[4]arene macrocycles, several architectures featuring a different pattern of substitution on the upper rim, including functional groups able to undergo acid dissociation, were synthesized (Figure 1). The self-assembly capability in polar solutions of amphiphilic resorc[4]arenes **R1**, **R2,** and **R3**, containing on the upper rim ester, carboxyl, and hydroxyl groups, respectively, were explored by UV-visible spectroscopy. Based on the ionization properties of **R2** and **R3**, we demonstrated that, by varying the pH values, the supramolecular assembly of resorc[4]arene-based systems can be driven in polar solutions paving the way for the design and construction of new drug formulations.

## 2. Results and Discussion

**Figure 2:**
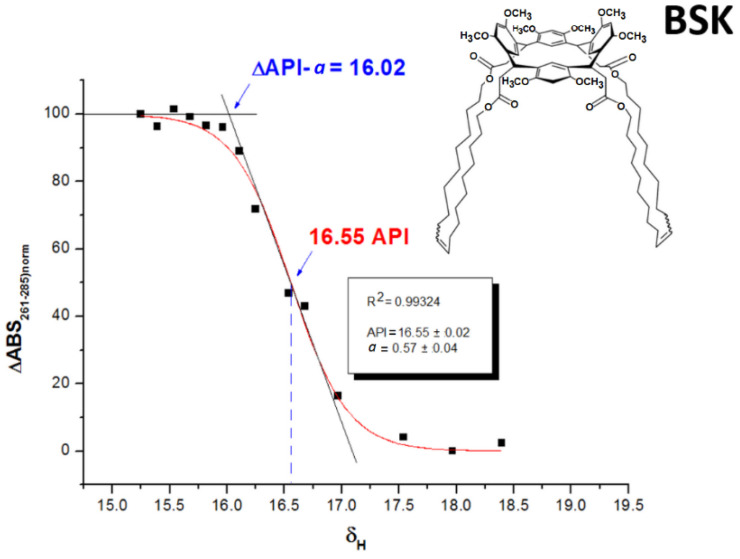
Sigmoidal aggregation profile of **BSK** as obtained in THF/water solvent system. The **API** parameter estimated for **BSK** from linear fitting of sigmoidal plot of **ΔABS_350–400_** vs. **δ_H_** is reported.

The construction of supramolecular assemblies by using amphiphilic molecular species is one of the most promising employable approaches to deliver hydrophobic pharmaceutically active compounds in physiological fluids. In principle, a suitable modulation of the self-assembly process can be effectively achieved in polar solvents by performing a proper chemical modification of the peripheral portion of the selected host, in terms of number and/or type of the hydrophilic groups. As such, the exploitation of supramolecular assembly of resorc[4]arenes represents a key approach to encapsulate hydrophobic bioactive compounds into their wide lipophilic and relatively flexible cavity-shaped architecture. In a previous study, we synthesized a cavity-shaped resorc[4]arene resembling a basket (**BSK**, Figure 2) via a ring closing metathesis reaction, and we investigated its self-aggregation propensity by UV-visible spectroscopy (Figure 2) [29]. To this aim, we developed a set of physical descriptors that, together, allowed us to calculate the hydrophilic−hydrophobic balance of the macrocycle. In this context, the Hildebrand polarity index (**δ_H_**) was employed as an indicator of the macrocycle affinity to the corresponding solvent system; a specific parameter, namely **API**, which corresponds to the **δ_H_** of the solvent mixture at which a self-aggregation process has reached the 50%, was used to reflect in a quantitative fashion the hydrophilic–hydrophobic nature of the molecule and, thus, its amphiphilicity [29].

The **BSK** resorcarene demonstrated a clear propensity to undergo self-aggregation in THF/water solvent systems. The aggregation phenomenon begins when the solvent composition shows a **δ_H_** value of 16.0, i.e., THF/water = 52:48 (*v*/*v*), and stops when **δ_H_** is about 17.1, i.e., THF/water = 44:56 (*v*/*v*) [29]. Specifically, the **API** index of **BSK** self-aggregation corresponded to a **δ_H_** = 16.55 (kcal × dm^3^)^1/2^ in the THF/water composition of 48/52 (*v*/*v*) [29]. The moderate **API** value found for **BSK** suggests that the hydrophobic nature of the macrocycle largely overcomes the hydrophilic one. In general, the linear relation between **δ_H_** and the water percentage of the THF/water mixture can be expressed by the following regression line achieved for the plot **δ_H_** vs. **H_2_O%** (**δ_H_** values have been obtained by the linear combination shown here: **H_2_O%** × **δ_H-of-H_2_O_** + **THF%** × **δ_H-of-THF_**, with the Hildebrand polarities **δ_H-of-H_2_O_** and **δ_H-of-THF_** amounting to 23.4 and 9.1, respectively) [35]:

**δ_H_** = 0.1435 × **H_2_O%** + 9.0929 (1)

**Figure 3:**
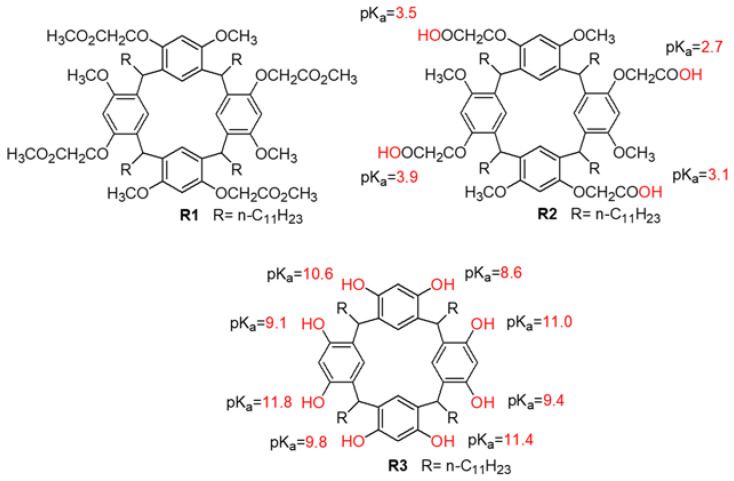
Structure of amphiphilic resorc[4]arenes **R1**, **R2,** and **R3**, and pK_a_ of acid groups calculated by Marvin program.

Based on this evidence, by increasing the hydrophilic character of the resorc[4]arene macrocycles through their upper rim chemical modification, the **API** parameter, as well as the range when the self-aggregation process occurs, should undergo a progressive shift towards greater values of **δ_H_**, corresponding to solutions largely rich in water which are able to give rise to a more effective solvation. Accordingly, we decided to synthesize three resorc[4]arene derivatives (**R1**, **R2,** and **R3**, Figure 3), featuring the same four alkyl chains in the lower rim, but different upper rim functionalization, and to investigate their self-assembly tendency by UV-visible spectroscopy. With respect to **R1** which contains four methyl ester groups, the **R2** and **R3** macrocycles own ionizable functions in their hydrophilic portion (i.e., carboxyl or phenolic groups). As predicted by theoretical pK_a_ values calculated through the Marvin program [36] (Figure 3), the degree of deprotonation of such groups can be finely modulated by the employment of the THF/water mixtures at a fixed pH value X of the aqueous component (pH_X_). Accordingly, to perform the self-assembly investigation of the ionizable resorc[4]arenes, the pH_X_ was set by using a suitable buffer solution, i.e., THF/(buffer-pH_X_) mixtures.

### 2.1. Synthesis of Amphiphilic Resorc[4]arene Macrocycles

**Scheme 1:**
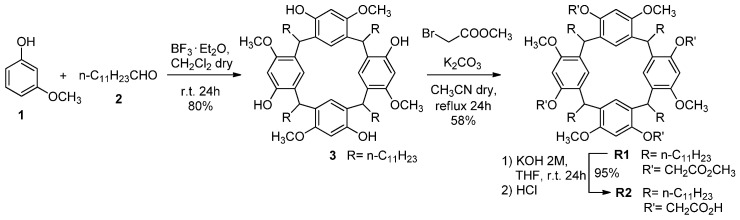
Synthesis of amphiphilic resorc[4]arenes **R1** and **R2**.

With the aim of constructing systems for pH-induced self-assembly of amphiphilic resorc[4]arenes, we introduced in the resorc[4]arene macrocycle scaffold four long non-polar hydrocarbon chains in the lower rim and polar groups in the upper rim. Resorcarene **R3** was prepared according to the literature [37,38]. Tetramethoxyresorcarenes (3) and **R1** were obtained by slight modifications of the synthetic procedures reported by Li et al [39]. The synthetic route to resorc[4]arenes **R1** and **R2** is reported in Scheme 1. Compound **3** was obtained by a tetramerization reaction of 3-methoxyresorcinol (1) with dodecanal (2). Successively, the phenol groups of resorcarene **3** were functionalized with methyl bromoacetate in the presence of potassium carbonate as a base, to obtain resorcarene **R1**, which bears methyl ester moieties in the upper rim. Finally, the ester functionalities of **R1** were hydrolyzed with 2 M of potassium hydroxide and then the solution was acidified with hydrochloric acid to obtain the resorc[4]arene tetraacid **R2**. All these ^1^H NMR and ^13^C NMR spectroscopical data were identical to the literature for compounds **3** and **R1** [39]. Compound **R2**, which was unknown, has been fully characterized by NMR and HRMS.

**Figure 4:**
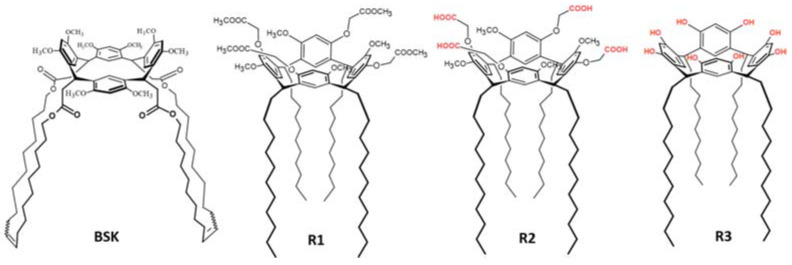
3D chemical structure of **BSK** and of amphiphilic resorc[4]arenes **R1**, **R2,** and **R3**.

For all the resorc[4]arenes, the ^1^H and ^13^C NMR spectral data are featured by the presence of single signals for equivalent internal and external aromatic protons and carbons, suggesting a cone conformation with C4v symmetry in solution (Figure 4). Accordingly, in addition to having a greater hydrophilic character, resorc[4]arenes **R1–R3** are less pre-organized and are more flexible systems with respect to **BSK**, in which a flattened cone conformation occurs for the presence of the two cyclic alkenes (Figure 4).

### 2.2. The Self-Association of Resorc[4]arene **R1** in THF/Water Solution

**Figure 5:**
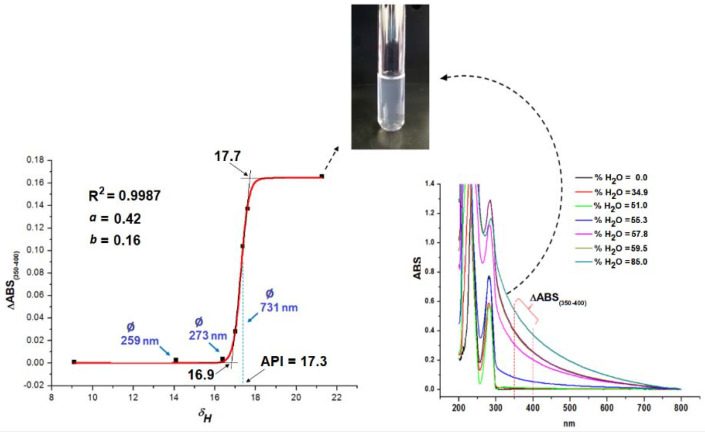
On the **left**, the sigmoidal aggregation profile of resorc[4]arene **R1** as obtained in THF/water solvent system. Regression analysis to fit the experimental data was performed according to Equation (2). On the **right**, the UV−visible spectra of resorc[4]arene **R1** (3.2 × 10^−5^ M) in different THF/water solvent systems.

The resorc[4]arene **R1**, which features similar structure to **R2** but endowed with a non-ionizable upper rim (-COOCH_3_ in place of -COOH), was used as a reference system to compare its self-assembly behavior with that of the ionizable resorc[4]arenes. Accordingly, we investigated the aggregation propensity of resorc[4]arene **R1** in THF/water mixtures by varying progressively the non-polar and polar solvent components in the **δ_H_** range from 9.1 (100% of THF) to 21.3 (THF/water = 15:85, *v*/*v*). For this purpose, the concentration of **R1** was kept at a constant value of 3.2 × 10^−5^ M. The self-association process was monitored by registering the changes in the UV-absorbance difference at the wavelengths of 350 and 400 nm (**ΔABS_350–400_**) as a function of the **δ_H_** value of the corresponding solvent system. The scatter plot of **ΔABS_350–400_** vs **δ_H_** was further fitted by using the following equation [29,40]:

**ΔABS_350–400_** = b/(1 + 10^(−(**δH** − **API**)/^^a^) (2)

with b and a parameters representing the maximum value assumed by **ΔABS_350–400_** and the slope of the sigmoidal curve, respectively. As depicted in Figure 5, the self-aggregation of **R1** is featured by an **API** value of 17.3 which corresponds to a water amount of 57.3% (with a = 0.42). This means that the resorc[4]arene assembly starts approximately when the THF/water composition yields **δ_H_** =16.9 (54.6% of water) and stops when **δ_H_** =17.7 (60.1% of water composition). At the end of the **R1** self-association process, a suspension with visible turbidity is formed (Figure 5). Although **R1** does not possess ionizable groups, to further characterize the structure in terms of lipophilic/hydrophilic balance of the macrocycle and to allow the comparison of its lipophilicity with that of resorc[4]arenes **R2** and **R3**, the distribution coefficient in the logarithmic form, Log(D), was calculated through the Marvin program [36]. In general, Log(D) is a widely used descriptor measuring the lipophilicity of ionizable biologically active compounds, where the partition in two immiscible solvents (octan-1-ol/water) is a function of the pH. Lower values of Log(D) correspond to structures endowed with higher aqueous solubility. Specifically, the Log(D) value of the resorc[4]arene **R1** was established to be 21.8.

### 2.3. The Self-Association of Ionizable Resorc[4]arenes **R2** and **R3** in THF/(Buffer-pH_X_) Solution

**Figure 6:**
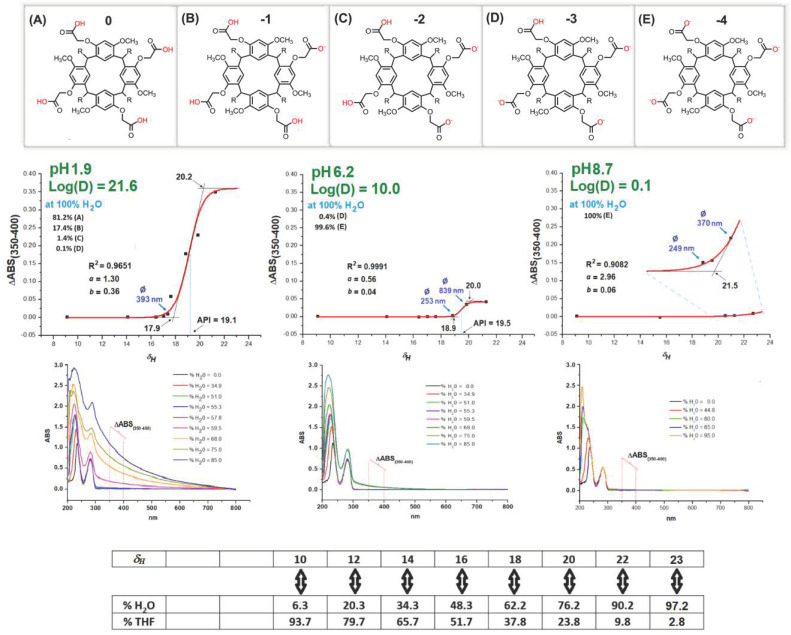
The sigmoidal aggregation profile and the corresponding UV-visible spectra of resorc[4]arene **R2** as obtained in THF/water solvent systems at fixed pH values of the aqueous component. Regression analysis to fit the experimental data was performed according to Equation (2). Subfigures (**A**–**E**) represent the deprotonation states of **R2** at the analyzed pH values of 1.9, 6.2 and 8.7 (the relevant percentages are shown next to the respective aggregation plots).

Due to the presence of four carboxyl groups in the upper rim, the **R2** degree of lipophilicity can be modulated in the Log(D) range from 21.6 to −5.2 by inducing the formation of the ionized forms at different pH values (in the range of 2.0–11.4). Accordingly, to investigate the self-assembly behavior of resorc[4]arene **R2**, the UV-visible spectroscopic analysis was performed by using the THF/(buffer-pH_X_) mixtures. To establish the final pH value (i.e., apparent pH) in the resulting THF/(buffer pH_X_) solution, the THF effect was experimentally measured up to its total amount of 50% in the mixture (see Figure S1). Specifically, as highlighted in Figure S1, the pH variation in the aqueous solution was overall rather modest reaching the maximum deviation at pH 8.4 (ΔpH = 0.54 units). To explore the influence of the **R2** deprotonation degree on the **δ_H_** ranges at which the self-assembly process begins and finishes, three different pH values (i.e., 1.9, 6.2, and 8.7) of the aqueous component employed in the THF/(buffer-pH_X_) mixtures were chosen. The ratio between the non-polar and the polar components of the solvent system was progressively varied in the **δ_H_** range from 9.1 (100% THF) to 21.3 (15% THF/85% water), when using buffer-pH_1.__9_ and buffer-pH_6.2_ solutions, and in the **δ_H_** range from 9.1 (100% THF) to 22.7 (5% THF/95% water), when using a buffer-pH_8.7_ solution. In all cases, the **R2** concentration was maintained at a fixed value of 3.0 × 10^−5^ M. The self-aggregation plots of **R2**, registered as a function of the THF/(buffer pH_X_) mixtures at the three above-mentioned pH values, are reported in Figure 6. By using a buffer-pH1.9 solution, the ionization of the resorc[4]arene **R2** is substantially suppressed and the Log(D) value accounts for 21.6. The total charge on the upper rim of **R2** was estimated to be −0.2, corresponding to the following distribution of each unionized and ionized species in water: 85% of the uncharged form; 14% of the mono-anionic form; and 1% of the di-anionic form. In the THF/(buffer-pH_1.__9_) mixture, the aggregation process of **R2** starts at **δ_H_** = 17.3 (57.3% of water) and stops at **δ_H_** = 20.9 (82.5% of water). By comparing the self-assembly of **R2** with that of **R1**, the more hydrophilic resorc[4]arene **R2** (Log(D) = 21.6 vs Log(D) = 21.8) begins the aggregation at a little bit greater **δ_H_** value (**δ_H_** = 17.3 vs. **δ_H_** = 16.9, corresponding to a difference of +2% in water), and completes the process to a higher **δ_H_** value (**δ_H_** = 20.9 vs. **δ_H_** = 17.7, corresponding to a difference of +23% in water).

**Figure 7:**
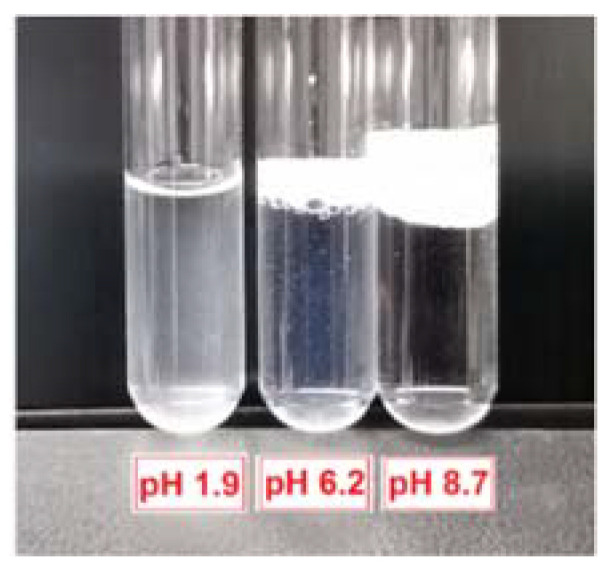
Turbid solution of **R2** at pH = 1.9; clear solution of **R2** at pH = 6.2 with some foam at liquid surface; clear transparent solution of **R2** at pH = 8.7 with consistent formation of a foam at the liquid surface.

When the aggregation process of **R2** was performed at the higher pH values (i.e., 6.2 and 8.7), marked changes in the **δ_H_** values, as well as in the **API** and a parameters, were found. In particular, by employing a buffer pH 6.2 solution, the self-assembly of **R2**, featuring an estimated Log(D) of 10.0, is comprised in the **δ_H_** range from 18.9 to 20.0, with **API** and a parameters of 19.5 and 0.56, respectively. At the end of this aggregation process, the solution appears slightly turbid, with the self-assembled molecules of **R2** showing a surfactant action evidenced by the formation of a small foam layer (Figure 7). When the self-aggregation process of **R2** was carried out by using the THF/(buffer-pH_8.__7_) solvent mixture, more drastic changes on the **API** and a parameters, as well as on the ***δ_H_*** value at which the assembly starts, were observed. In a buffer-pH_8.__7_ solution, the resorc[4]arene **R2** is characterized by a Log(D) of 0.1, and it is completely deprotonated. As such, the process is triggered when **δ_H_** reaches the value of 20.5 (i.e., 86% of water), with the **API** and a parameters assessed equal to 22.5 (i.e., 94% of water composition) and 2.17, respectively. In these conditions, unlike in buffer-pH_1.__9_ and buffer-pH_6.__2_ systems, at the end of the self-assembly process a clear solution appears, featured by a very low **ΔABS_350–400_** value of 0.01 (about ten times lesser than that registered for the aggregated form of **R2** at pH = 6.2). The self-assembled molecules of **R2** show a strong surfactant action, as evidenced by the formation of a thick layer of foam (Figure 7). These results suggest that, in such an experimental condition, the resorc[4]arene **R2** might act as an effective molecular shuttle of hydrophobic structures. Interestingly, the Log(D) value of 0.1 assessed for **R2** at pH = 8.7 corresponds to the one owned by the stearic acid at pH = 11.5 and by the palmitic acid at pH = 10.5 (values calculated by Marvin [36]). The sodium salts of these fatty acids, which are the common components of natural soaps, are typically characterized in water by pH values close to 11. Thus, resorc[4]arene **R2** at a pH of around 9 is featured by a similar lipophilic/hydrophilic balance to that of components of natural soaps.

**Figure 8:**
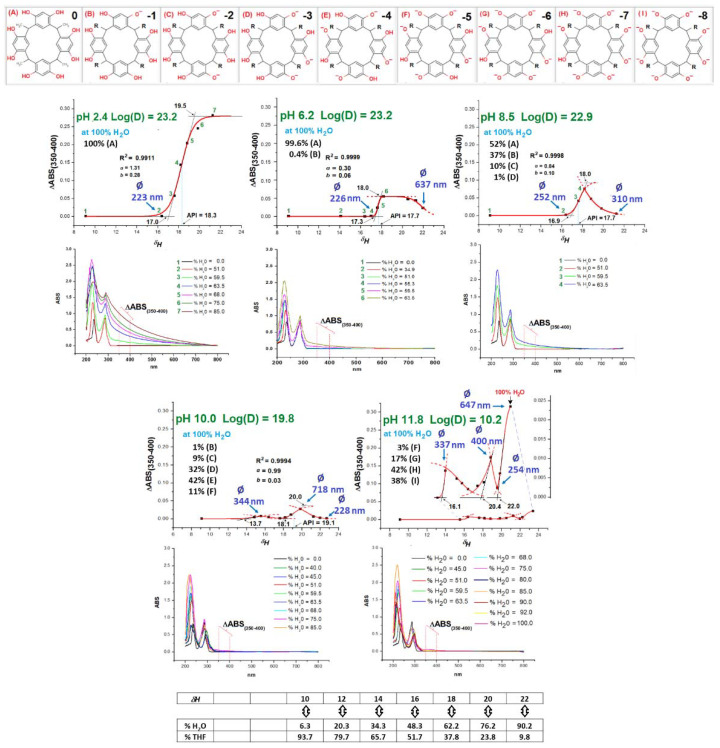
The sigmoidal aggregation profiles and the corresponding UV-visible spectra of resorc[4]arene **R3** as obtained in THF/water solvent systems at fixed pH values of the aqueous component. Regression analysis to fit the experimental data was performed according to Equation (2). Subfigures (**A**–**I**) represent the deprotonation states of **R3** at the analyzed pH values of 2.4, 6.2, 8.5, 10.0 and 11.8 (the relevant percentages are shown next to the respective aggregation plots).

**Figure 9:**
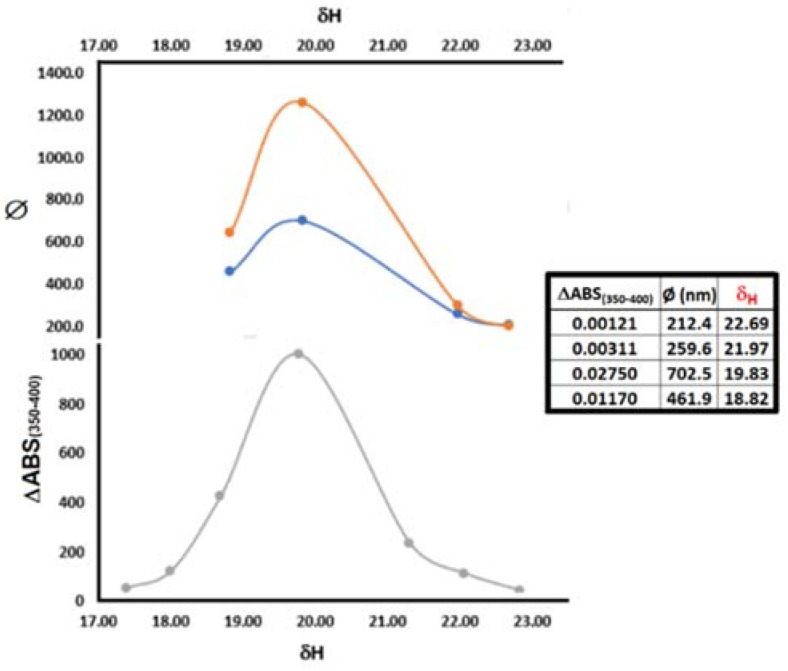
DLS profiles of **R3** aggregates in solutions prepared from a THF/(buffer-pH_10.0_) mixture: variation of Ø (blue) related to the **ΔABS_350–400_** changes at the same **δ_H_** range (gray); variation of Ø (orange) after one hour.

Further investigation was focused on the self-assembly behavior of resorc[4]arene **R3**, featuring eight ionizable phenolic groups on the upper rim. The aggregation process was monitored in the pH range from 2.4 to 11.8 by using specific THF/(buffer-pH_X_) mixtures (i.e., X = 2.4, 6.2, 8.5, 10.0, 11.8), in order to allow a selective modulation of **R3** hydrophilicity in response to an appropriate pH value. Accordingly, the Log(D) values of resorc[4]arene **R3** were assessed by the Marvin program [36] as a function of the selected pH (Figure 8) and the theoretical pK_a_ values of the phenolic groups (Figure 3), thus reflecting the different percentages in which **R3** is neutral or in the charged forms. The **ΔABS_350–400_** values plotted as a function of ***δ_H_*** for each THF/(buffer-pH_X_) mixture are collected in Figure 8. From the sigmoidal plots, an initial induction of **R3** self-aggregation is followed by a progressive disaggregation step, except for the THF/(buffer-pH_2.4_) mixture which preserves **R3** in its uncharged form. To experimentally explain this trend, Dynamic Light Scattering (DLS) measurements were carried out by analyzing the diameters Ø of the **R3** aggregates in solutions prepared from a THF/(buffer-pH_10.__0_) mixture in the **δ_H_** range from 18.8 to 22.7 (i.e., from 68% to 95% of water composition). As outlined in Figure 9, the variation of Ø (blue line) is perfectly related to the **ΔABS_350–400_** changes at the same **δ_H_** range (gray line). After one hour, the DLS measurements were performed on the same solutions, showing how the **R3** aggregates significantly increase in dimensions by a factor of 1.8 at the water composition of 75%, while to a lesser extent at 68% of water (Figure 8, orange line). Interestingly, the measured diameters of the **R3** aggregates are linearly related to the **ΔABS_350–400_** values at the same **δ_H_** index (R² = 0.9869), according to the following equation:

Ø = 18,532 × **ΔABS_350–400_** + 207.49 (3)

Within the **ΔABS_350–400_** range of 0.001–0.03, this equation was employed to estimate the variation in the aggregate size of the resorc[4]arenes **R1**, **R2,** and **R3** as a function of **δ_H_**. The **δ** of the most significant aggregates are indicated in the sigmoidal profiles of Figure 4, Figure 5 and Figure 7. The propensity of the macrocycle to self-assembly with the formation of colloidal aggregates is clearly demonstrated from the aggregation plots of **R3**. At higher pH and **δ_H_** values (water percentages greater than 85%), their diameters are lesser than 300 nm, giving rise to lyophilic colloids and thus to clear solutions. Nevertheless, by using a THF/(buffer-pH_11.__8_) mixture at **δ_H_** > 20 (water composition greater than 90%), the diameter of the colloid system exceeds the above limit, reaching the value of 647 nm in 100% of buffer and leading to a perfectly clear solution. Similarly to resorc[4]arene **R2** in the THF/(buffer-pH_8.__7_) solvent system at **δ_H_** greater than 20.5, the **R3** solutions at specific pH and **δ_H_** values might favor the solubilization of hydrophobic compounds.

### 2.4. Lyophilic Colloids Based on Self-Aggregated Resorc[4]arenes **R2** and **R3**

**Figure 10:**
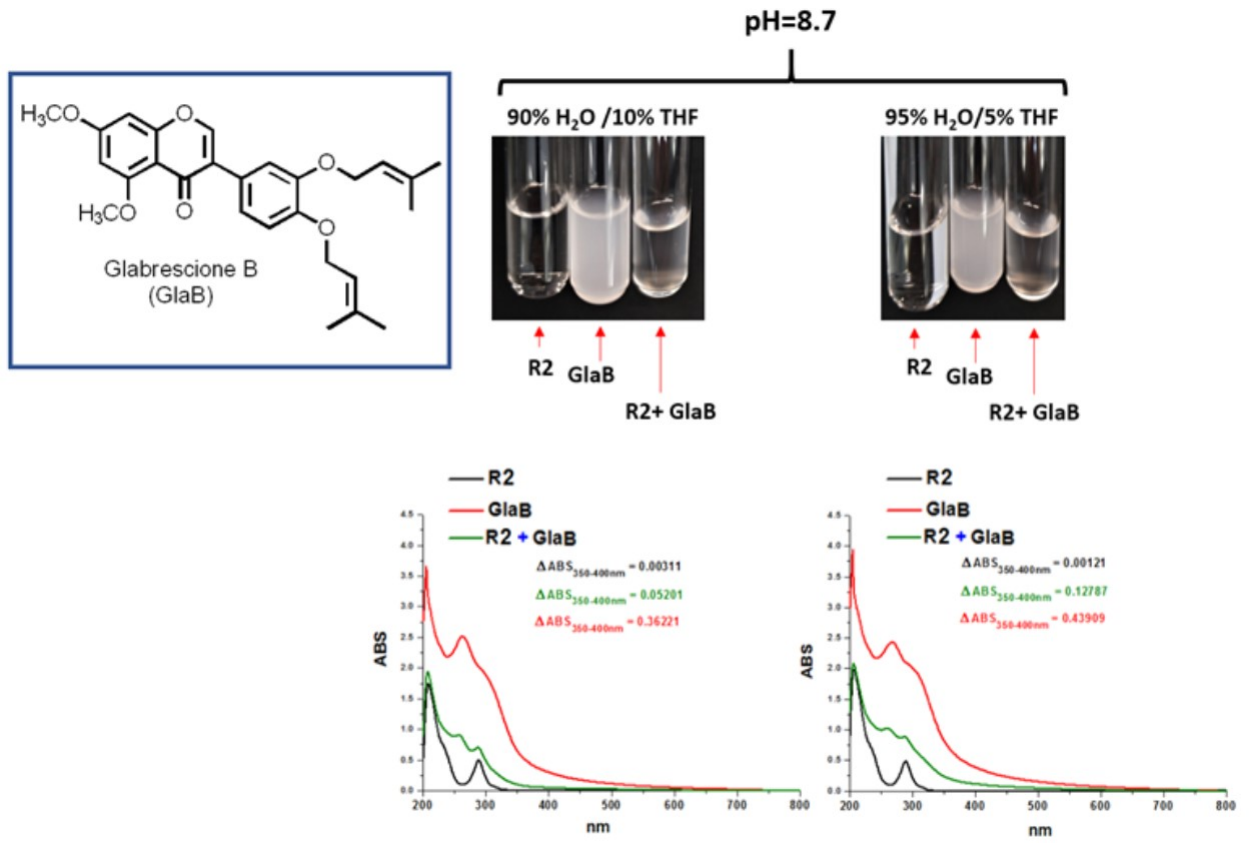
The ability of lyophilic colloids based on resorc[4]arene **R2** to encapsulate GlaB by using a THF/(buffer-pH_8.__7_) mixture at different **δ_H_** values.

The ability of lyophilic colloids based on resorc[4]arenes **R2** and **R3** to capture hydrophobic compounds in wide polar media was investigated towards Glabrescione B (GlaB, Log(D) = 5.14) (Figure 9), a naturally-occurring isoflavone which proved to be a good preclinical candidate for the treatment of Hedgehog (Hh) dependent tumors [41,42,43,44]. Based on the above-mentioned results, the self-aggregation process of **R2** was induced in its completely deprotonated form by using a THF/(buffer-pH_8.7_) mixture at **δ_H_** values of 21.97 and 22.69 (corresponding to 90% and 95% of water composition, respectively). As showed in Figure 10, while GlaB alone gives rise to cloudy suspensions in both selected **δ_H_** conditions, by using the **R2** lyophilic colloids, the turbidity, although present, appears strongly reduced.

**Figure 11:**
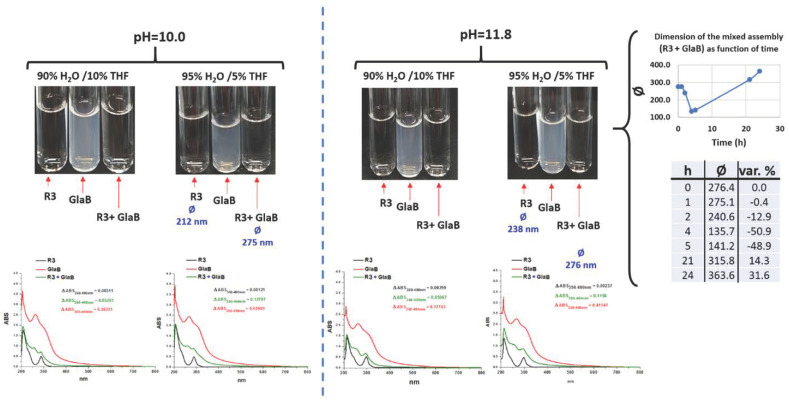
The ability of lyophilic colloids based on resorc[4]arene **R3** to act as a molecular shuttle of GlaB by using THF/(buffer-pH_10.__0_) and THF/(buffer-pH_11.8_) mixtures at different **δ_H_** values.

The observed behavior of resorc[4]arene **R3** was rather different. The aggregation test of GlaB alone and in the presence of **R3** was performed by employing THF/buffer mixtures featuring different **δ_H_** values: (a) 19.83, 21.97, and 22.69, with the use of buffer-pH_10.__0_ solution; (b) 21.97 and 22.69, with the use of buffer-pH_11.8_ solution. As outlined in Figure 11, by using an equimolar concentration of GlaB and **R3** (3.0 × 10^−5^ M), clear colloidal solutions at both the analyzed pH values were obtained. Coherently, the aggregate dimensions established by the DLS measurements indicate that the lyophilic colloids (**R3** + GlaB) at **δ_H_** = 22.69 reach diameters very close to the ones measured for the self-aggregated **R3**, at both the pH values of 10.0 and 11.8. In addition, the dimensional stability of lyophilic colloids (**R3** + GlaB) in the THF/(buffer-pH_11.8_) solvent system at **δ_H_** = 22.69 was analyzed over time by DLS measurements (Figure 11). The **R3**-GlaB aggregates were stable within the first hour (h), characterized by a diameter of 276 nm. Later (4 h), a reduction occurred, reaching a **δ** of 136 nm, and a further increment towards the value of 400 nm was observed within 24 h.

## 3. Materials and Methods

### 3.1. Synthesis of Resorc[4]arenes

General remarks: melting points were recorded with a Büchi melting point B-545 and are not corrected. The ^1^H and ^13^C NMR spectra have been acquired with a Bruker Avance 400 spectrometer operating at 400.13 and 100.6 MHz, respectively, at 300 K in CDCl_3_ or DMSO-d_6_, using 5 mm diameter glass tubes. Chemical shifts were expressed in ppm and coupling constants (J) in hertz (Hz), approximated to 0.1 Hz. The residual solvent peak was used as an internal reference for ^1^H and ^13^C NMR spectra. Data for ^1^H NMR are reported as follows: chemical shift, multiplicity (br = broad, ovrlp = overlapped, s = singlet, d = doublet, t = triplet, q =quartet, m = multiplet, dd = double doublet), coupling constant, and integral. Spectra were processed with the program MestReNova version 6.0.2-5475, FT and zero filling at 64 K. High-resolution (HR) mass spectra were obtained using a Thermo Fischer Exactive mass spectrometer equipped with an ESI source and an Orbitrap analyzer: capillary temperature 275 °C, spray voltage 3.5 kV, sheath gas (N_2_) 10 arbitrary units, capillary voltage 65 V, and tube lens 125 V. Analytical TLC were performed using 0.25 mm Fluka F254 silica gel. The compounds on TLC were revealed by quenching fluorescence (at 254 and 365 nm) using a 4 W UV lamp. Otherwise, plates were stained with an acidic solution of p-anisaldehyde or a 10% phosphomolybdic acid solution in EtOH and heated (T = 120 °C). The product mixture purifications were carried out with silica column chromatography using Fluka 60 Å silica gel (0063−0200 mm, 70−230 mesh). Flash chromatography was performed using 200−400 mesh silica gel. Commercially available reagents were supplied by Sigma-Aldrich and used without further purification. Dry solvents were purchased from Sigma-Aldrich or dried by distillation. Resorcarene **R3** [37,38] and GlaB [45] were synthesized according to the literature. Yields of synthesized compounds are referred to chromatographically and spectroscopically pure compounds, unless otherwise stated.

### 3.2. Synthesis of Tetraundecanyl Tetra-O-methyl Resorc[4]arene (**3**)

Boron trifluoride etherate (2.3 g, 2 mL, 16.2 mmol) was added to a solution of 3-methoxyphenol (1) (1 g, 0.88 mL, 8.0 mmol) and dodecanal (2) (1.47 g, 1.76 mL, 8.0 mmol) in anhydrous dichloromethane (40 mL), and the reaction was kept under stirring at room temperature for 2 h. The reaction mixture was then washed with water (2 × 40 mL) and brine (1 × 40 mL). The organic layer was dried over anhydrous Na_2_SO_4_, and the solvent was removed under reduced pressure to give a dark red oil. The crude was crystalized from hot ethanol to give a reddish solid. The product was recrystallized from hot methanol to obtain pure compound **3** (0.687 g, 80% yield) as a pale pinkish solid. ^1^H NMR (400 MHz, CDCl_3_) δ 7.51 (s, 4H), 7.22 (s, 4H), 6.35 (s, 4H), 4.27 (t, J = 7.4 Hz, 4H), 3.83 (s, 12H), 2.19 (d, J = 6.7 Hz, 8H), 1.27 (M, 72H), and 0.89 (t, J = 6.3 Hz, 12H); ^13^C NMR (101 MHz, CDCl_3_) δ 153.7, 153.1, 124.9, 124.7, 123.8, 100.1, 77.5, 77.2, 76.8, 56.0, 34.1, 33.2, 32.1, 29.9, 29.9, 29.6, 28.2, 22.8, and 14.3.

### 3.3. Synthesis of Tetraundecanyl-tetra(methoxycarbonylmethoxyl)-tetra-O-methyl Resorc[4]arene (**R1**)

Methyl bromoacetate (0.225 mL, 0.364 g, 2.38 mol) was added to a stirred solution of resorc[4]arene 3 (0.554 g, 0.476 mmol) and K_2_CO_3_ (0.654 g, 4.76 mmol) in dry acetonitrile (65 mL), and the reaction mixture was heated at reflux for 24 h under inert atmosphere. Then, the reaction mixture was cooled down and the solvent was removed under reduced pressure. The residue was dissolved in dichloromethane (40 mL), and the organic layer was washed with 1 M HCl (10 mL), with water and brine. The organic layer was dried over anhydrous Na_2_SO_4_, and the solvent was removed under reduced pressure. The pure product **R1** was obtained as a solid (0.398 g, 0.274 mmol) in 58% yield and used without further purification. ^1^H NMR (400 MHz, CDCl_3_) δ 6.61 (s, 4H), 6.29 (s, 4H), 4.50 (t, J = 7.4 Hz, 4H), 4.21 (d, J = 15.9 Hz, 4H), 4.02 (d, J = 15.9 Hz, 4H), 3.77 (s, 12H), 3.61 (s, J = 5.5 Hz, 12H), 1.85–1.77 (m, 8H), 1.32–1.21 (ovrlp m, 18H), and 0.87 (t, J = 6.9 Hz, 12H); ^13^C NMR (101 MHz, CDCl_3_) δ 170.3, 155.7, 155.0, 128.4, 127.6, 126.5, 99.7, 77.5, 77.2, 76.8, 68.4, 55.6, 52.0, 50.9, 35.6, 34.8, 32.1, 30.1, 30.0, 29.9, 29.87, 29.83, 29.5, 28.2, 22.8, and 14.2.

### 3.4. Synthesis of Tetraundecanyl-tetra(hydroxycarbonylmethoxyl)-tetra-O-methyl Resorc[4]arene (**R2**)

A 2 M aqueous solution of potassium hydroxide (15 mL) was added to a solution of resorc[4]arene **R1** (0.400 g, 0.276 mmol) in THF (40 mL), and the reaction mixture was stirred for 24 h at room temperature. Then the solution was acidified with 2 M HCl (40 mL) and the THF was removed under reduced pressure. The white precipitate was filtered, washed with water, and dried under a vacuum at 80 °C for 3 h. Then it was dissolved in THF and the solution was filtered. The THF was removed under reduced pressure to give **R2** as a white powder (0.366 g, 0.262 mmol) in 95% yield. ^1^H NMR (400 MHz, DMSO-d_6_) δ 6.65 (s, 4H), 6.38 (s, 4H), 4.51 (t, J = 7.0 Hz, 4H), 4.42 (d, J = 16.1 Hz, 4H), 4.25 (d, J = 16.1 Hz, 4H), 3.57 (s, 12H), 1.69 (br s, 8H), 1.16 (s, 72H), and 0.79 (t, J = 6.7 Hz, 12H). ^13^C NMR (101 MHz, DMSO-d_6_) δ 170.50, 155.23, 154.27, 125.88, 125.30, 125.23, 98.43, 66.47, 55.47, 35.05, 34.11, 31.40, 30.69, 29.58, 29.35, 29.19, 28.84, 27.62, 22.14, and 13.82. ESI-HRMS: *m*/*z* [M−H]^−^ C_84_H_127_O_16_ requires 1391.9130, found 1391.9100; [M−2H]^−2^ C_84_H_126_O_16_ requires 695.4528, found 695.4524; and [M−3H]^−3^ C_84_H_125_O_16_ requires 463.2995, found 463.2993.

### 3.5. UV-Vis Spectroscopical Analyses

General remarks: all spectroscopic analyses were performed with the JASCO V-550 spectrometer with a Peltier thermostat at 25 °C using a quartz cuvette (cell length 1 mm). The HPLC grade THF (tetrahydrofuran) and H_2_O (water) were obtained from Sigma Aldrich, St. Louis, MO, USA.

### 3.6. Preparation of Solutions

Stock solution of **R1** (C_88_H_136_O_16_, Mw 1449.76 g/mol; 6.1 × 10^−3^ g, 4.2 × 10^−3^ mmol) at a concentration of 2.1 × 10^−4^ M in 20 mL of THF was prepared. Starting from this solution, the samples used for the UV spectrophotometric analysis were obtained with a different THF/H_2_O ratio (from 0% to 85% of water) having a final concentration of **R1** equal to 3.2 × 10^−5^ M and a final volume of 2 mL. The baseline was obtained with the same THF/H_2_O ratio as the samples. Stock solution of **R2** (C_84_H_128_O_16_, Mw 1392.92 g/mol; 5.6 × 10^−3^ g, 4.0 × 10^−3^ mmol) at a concentration of 2.0 × 10^−4^ M in 20 mL of THF was prepared. Starting from this solution, the samples used for the UV spectrophotometric analysis were obtained with a different THF/H_2_O ratio (from 0% to 85% of water) having a final concentration of **R2** equal to 3.0 × 10^−5^ M and a final volume of 2 mL. The baseline was obtained with the same THF/H_2_O ratio as the samples. Modifications of the pH were obtained by using an aqueous solution of phosphate buffer (NaH_2_PO_4_, Mw 119.98 g/mol, 8.2 × 10^−3^ g in 50 mL of H_2_O, 10^−3^ M and H_3_PO_4_, Mw 97.99 g/mol) at a different ratio. The baseline was obtained with the same THF/buffer phosphate ratio as the samples. Stock solution of **R3** (C_72_H_112_O_8_, Mw 1105.65 g/mol; 10.5 × 10^−3^ g, 9.5 × 10^−3^ mmol) at a concentration of 1.9 × 10^−4^ M in 20 mL of THF was prepared. Starting from this solution, the samples used for the UV spectrophotometric analysis were obtained with a different THF/H_2_O ratio (from 0% to 85% of water) having a final concentration of **R3** equal to 2.85 × 10^−5^ M and final volume of 2 mL. The baseline was obtained with the same THF/H_2_O ratio as the samples. Modifications of the pH at 11.78 were obtained by an aqueous solution of sodium tetraborate decahydrate (Na_2_B_2_O_7_·10 H_2_O, Mw 381.49 g/mol, 5.63 × 10^−2^ g in 100 mL of H_2_O, 1.5 × 10^−3^ M) and sodium hydroxide (NaOH, Mw 39.99 g/mol, 1 N). The baseline was obtained with the same THF/buffer borate ratio as the samples. A stock solution of GlaB (C_27_H_30_O_6_, Mw 450.5 g/mol; 3.6 × 10^−3^ g, 8.0 × 10^−3^ mmol) of 4 × 10^−4^ M in 20 mL of THF was prepared. Starting from this solution, the samples were diluted in THF in a ratio of 1:2 (final concentration 2 × 10^−4^ M) and used for the UV spectrophotometric analysis were obtained with a different THF/buffer phosphate (pH 11.82) ratio (from 0% to 85% of water) having a final concentration of GlaB equal to 3.0 × 10^−5^ M and a final volume of 2 mL. The baseline was obtained with the same THF/buffer phosphate ratio as the samples. Stock solution of guest (GlaB) and host (**R3**) in a ratio of 1:1 was prepared in THF. Starting from this solution, the samples that were used for the UV spectrophotometric analysis were obtained with a different THF/buffer phosphate (pH 11.85) ratio (from 0% to 85% of water) having a final concentration of GlaB and **R3** equal to 3.0 × 10^−5^ M and a final volume of 2 mL. The baseline was obtained with the same THF/buffer phosphate ratio as the samples. Starting from the stock solutions of **R3** and GlaB at a concentration of 1.2 × 10^−3^ M, 0.100 mL of sample was taken and solubilized in 0.100 mL of THF and 1.8 mL of buffer to obtain the concentration of water at 90%. A 0.100 mL of solubilized sample in 1.9 mL of buffer was used to obtain a 95% water concentration. In both cases the sample had a concentration of 6 × 10^−5^ M. A 1 mL of solution of **R3** was added to a 1 mL of solution of GlaB to give a final volume of 2 mL, with a final equimolar concentration of the compounds equal to 3 × 10^−5^ M.

### 3.7. DLS Analysis

The size and z potential values of resorc[4]arene **R3** were measured by using a 90Plus/BI-MAS ZetaPlus multiangle particle size analyzer (Brookhaven Instruments Corp., Holtsville, NY, USA). For size measurements, the autocorrelation function of the scattered light was analyzed assuming a log Gaussian distribution of the vesicle size. The mean size and polydispersity index have been obtained. The z potential values were calculated from the electrophoretic mobility by means of the Helm-holtz-Smoluchowski relationship.

## 4. Conclusions

In conclusion, we carried out a detailed characterization of the self-assembly process of amphiphilic resorc[4]arene-based architectures featuring long aliphatic side chains and a different pattern of substitution on the upper rim, including functional groups able to undergo acid dissociation. Based on the hydrophilic features and the ionization properties of the upper rim of the macrocycles, these amphiphiles revealed a strong propensity to self-assembly in a specific THF/water composition. The combination of theoretical calculations with the experimental results highlighted that the supramolecular assembly of ionizable resorc[4]arenes is strictly dependent on the pH values, when using solutions largely rich in water (i.e., 10% THF/90% H_2_O and 5% THF/95% H_2_O), leading to the formation of lyophilic colloids with characteristic diameters. Based on these properties, we demonstrated that the resorc[4]arene-based systems can entrap the poorly water-soluble isoflavone GlaB, most probably due to inclusion complexation between the guest molecules and the hydrophobic alkyl chains of the macrocycles. The next steps of the study will be: (i) the NMR investigation to characterize the inclusion complexation more deeply; (ii) the design of novel amphiphilic architectures featuring ionizable functional groups with improved ability to supramolecular self-assemble in water at specific pH values. In addition, we will investigate the in vitro bioactivity of GlaB-resorcarene aggregates in the anticancer efficiency towards the Hh-dependent tumors.

## Data Availability

Not applicable.

## Supplementary Materials

The following are available online at https://www.mdpi.com/article/10.3390/ijms222111785/s1, Figure S1: pH variations of THF/water solutions depending on the mixture composition and different prefixed pH values, Figure S2: ^1^H NMR spectrum of compound **3** (CDCl_3_, 400 MHz), Figure S3: ^13^C NMR spectrum of compound **3** (CDCl_3_, 101 MHz), Figure S4: ^1^H NMR spectrum of compound **R1** (CDCl_3_, 400 MHz), Figure S5: ^13^C NMR spectrum of compound **R1** (CDCl_3_, 101 MHz), Figure S6: ^1^H NMR spectrum of compound **R2** (DMSO-d_6_, 400 MHz), Figure S7: ^13^C NMR spectrum of compound **R2** (DMSO-d_6_, 101 MHz), Figure S8: ESI-HRMS spectrum of compound **R2**, Table S1: **R3** pH 9.96 from 68% to 95% H_2_O, Table S2: **R3** + GlaB pH 11.8, 95% H_2_O, Table S3: **R1**, Table S4: **R2** pH 6.21, Table S5: **R2** pH 1.91, Table S6: **R2** pH 8.70, Table S7: **R3** pH 6.21, Table S8: **R3** pH 2.41, Table S9: **R3** pH 8.52, Table S10: **R3** pH 10.0, Table S11: **R3** pH 11.8.
