# Supplementary material for: Exploring the Assembly of Resorc[4]arenes for the Construction of Supramolecular Nano-Aggregates
Source: Int J Mol Sci. 2021 Oct 29;22(21):11785. doi: 10.3390/ijms222111785 (PMC8584166; doi:10.3390/ijms222111785)
Supplement: Supplementary file 1 [file ijms-22-11785-s001.zip › ijms-1403910-supplementary.pdf]

Exploring the Assembly of Resorc[4]arenes for the Construction of Supramolecular Nano-aggregates

Fabio Buonsenso [1], Francesca Ghirga [1], Isabella Romeo [1, 2], Gabriella Siani [3], Serena Pilato [3], Deborah Quaglio [1, *], Marco Pierini [1, *], Bruno Botta [1] and Andrea Calcaterra [1]

[1] Department of Chemistry and Technology of Drugs, "Department of Excellence 2018−2022", Sapienza University of Rome, P. le Aldo Moro 5, 00185, Rome, Italy

[2] Center for Life Nano and Neuroscience Italian Institute of Technology, Viale Regina Elena 291, 00161, Rome, Italy

[3] Department of Pharmacy, University of Chieti "G. D'Annunzio", via dei Vestini 31, 66013, Chieti, Italy

Corresponding author e-mail: deborah.quaglio@uniroma1.it; marco.pierini@uniroma1.it

Table of contents

1. pH variations 2

2. ^{1}H and ^{13}C NMR spectra 3

  2.1 Compound **3** 3

  2.2 Compound **R1** 4

  2.3 Compound **R2** 5

3. ESI-HRMS of **R2** 6

4. UV-visible experiments and DLS measurements 7

  4.1 Table S1 7

  4.1 Table S2 7

  4.1 Table S3 7

  4.1 Table S4 8

  4.1 Table S5 8

  4.1 Table S6 9

  4.1 Table S7 9

  4.1 Table S8 10

  4.1 Table S9 10

  4.1 Table S10 11

  4.1 Table S11 12

* Corresponding author. e-mail: deborah.quaglio@uniroma1.it; marco.pierini@uniroma1.it

1. pH variations

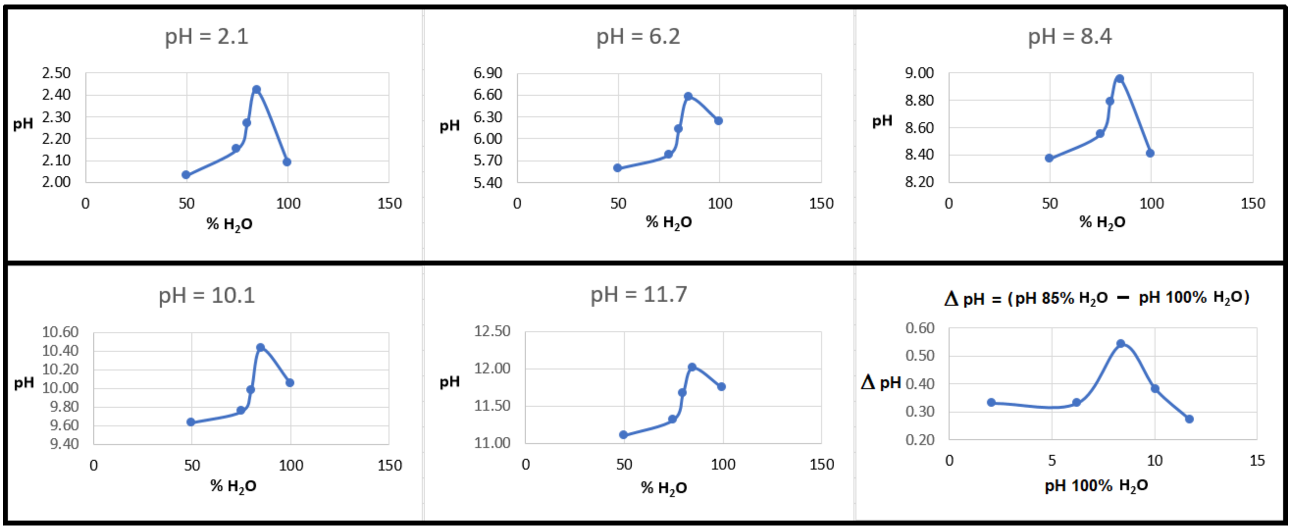

Fig. S1. pH variations of THF/water solutions depending on the mixture composition and different prefixed pH values.

2. ^{1}H and ^{13}C NMR spectra

Compound 3

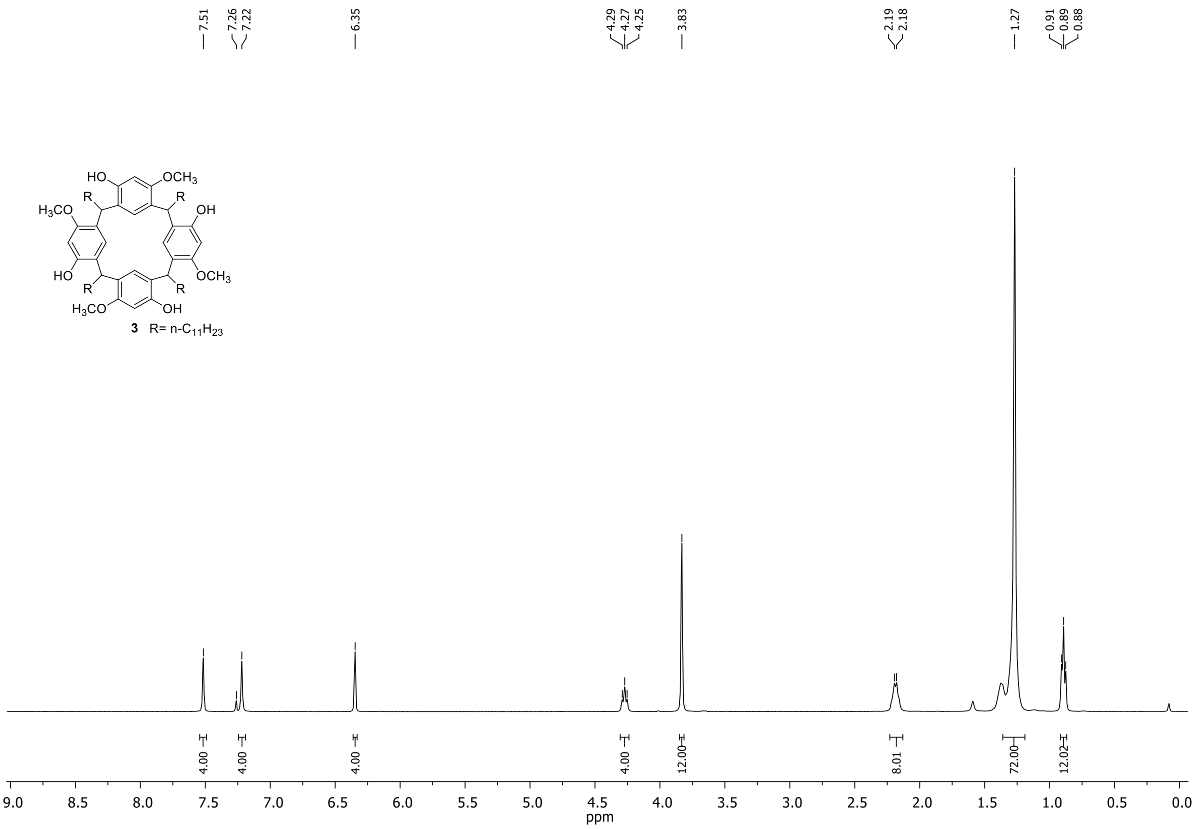

Fig. S2. ^{1}H NMR spectrum of compound **3** ($CDCl_3$, 400 MHz).

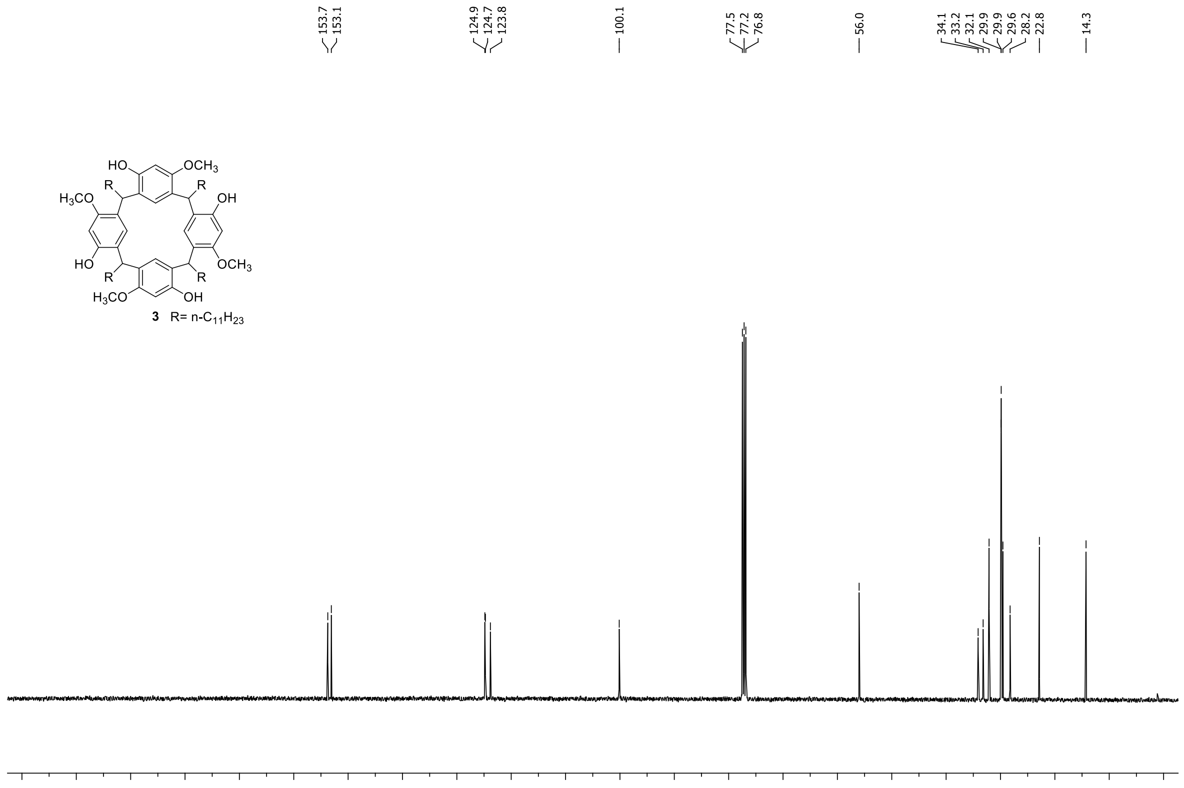

Fig. S3. ^{13}C NMR spectrum of compound **3** ($CDCl_3$, 101 MHz).

Compound R1

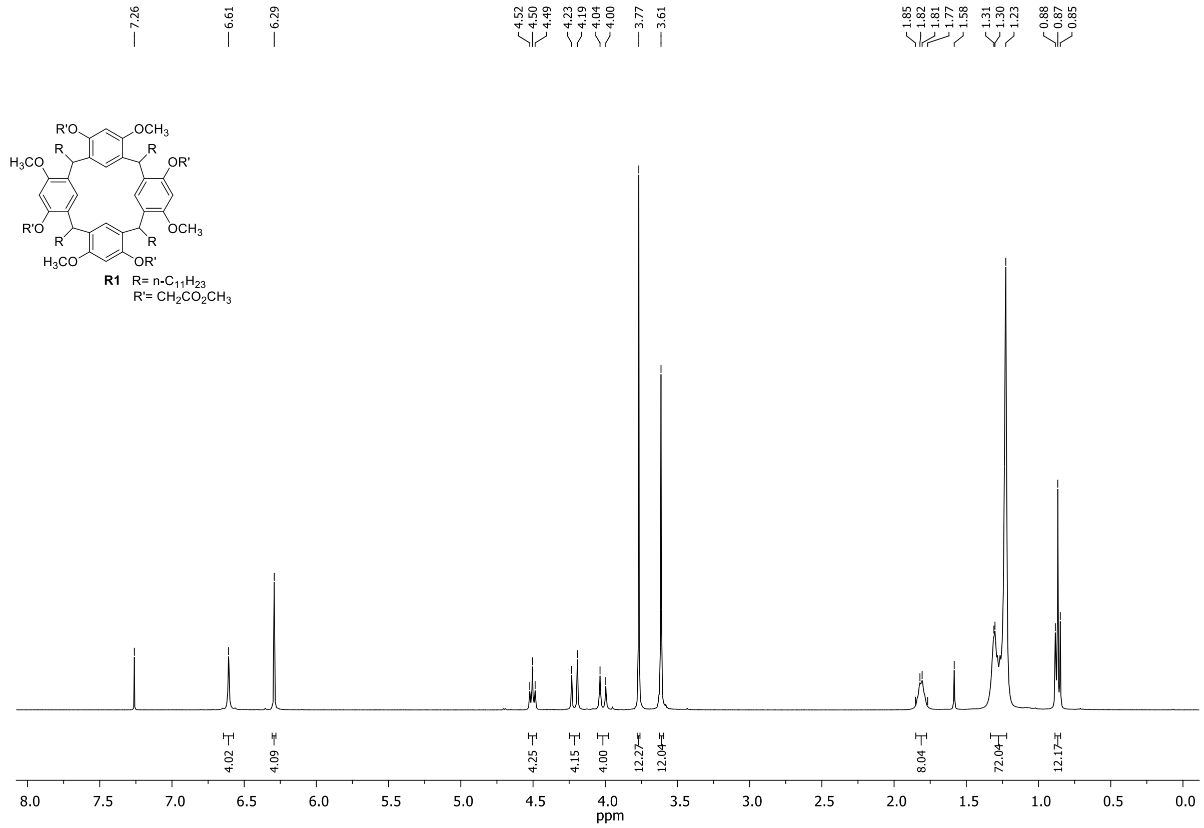

Fig. S4. ^{1}H NMR spectrum of compound **R1** ($CDCl_3$, 400 MHz).

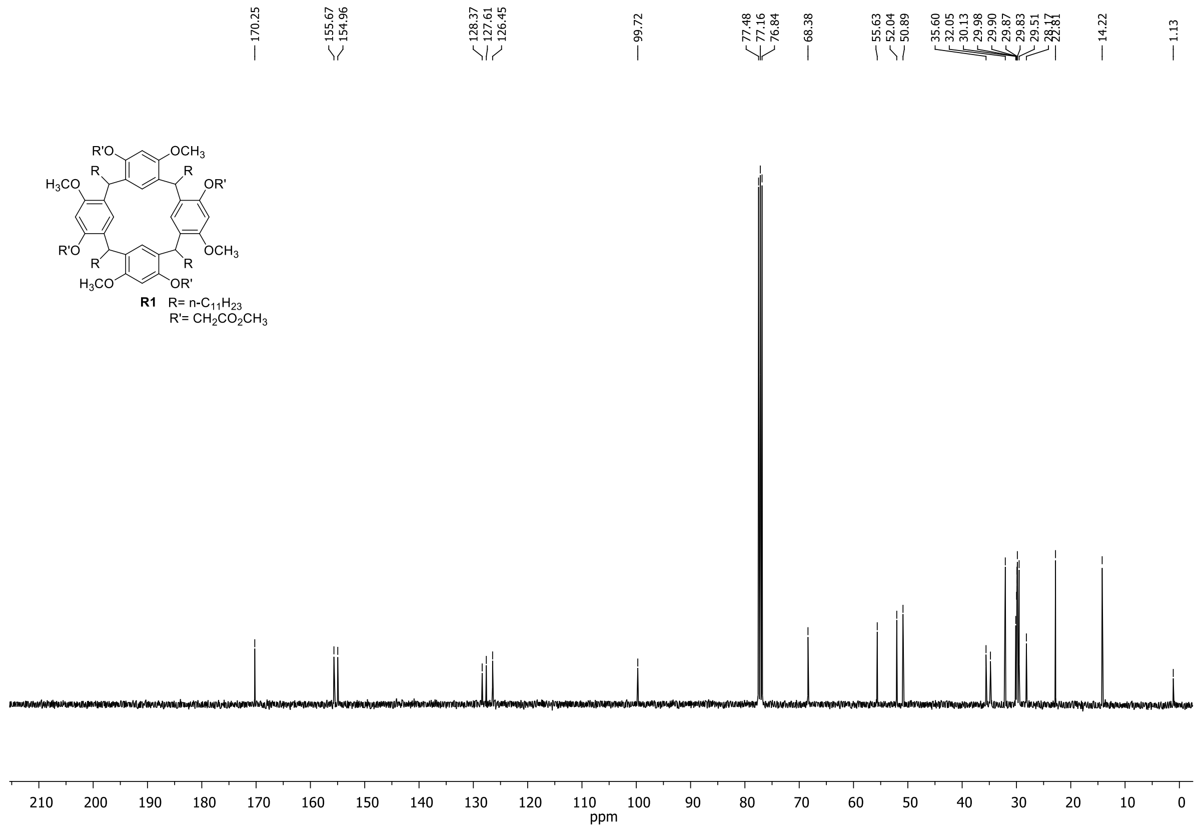

Fig. S5. ^{13}C NMR spectrum of compound **R1** ($CDCl_3$, 101 MHz).

Compound R2

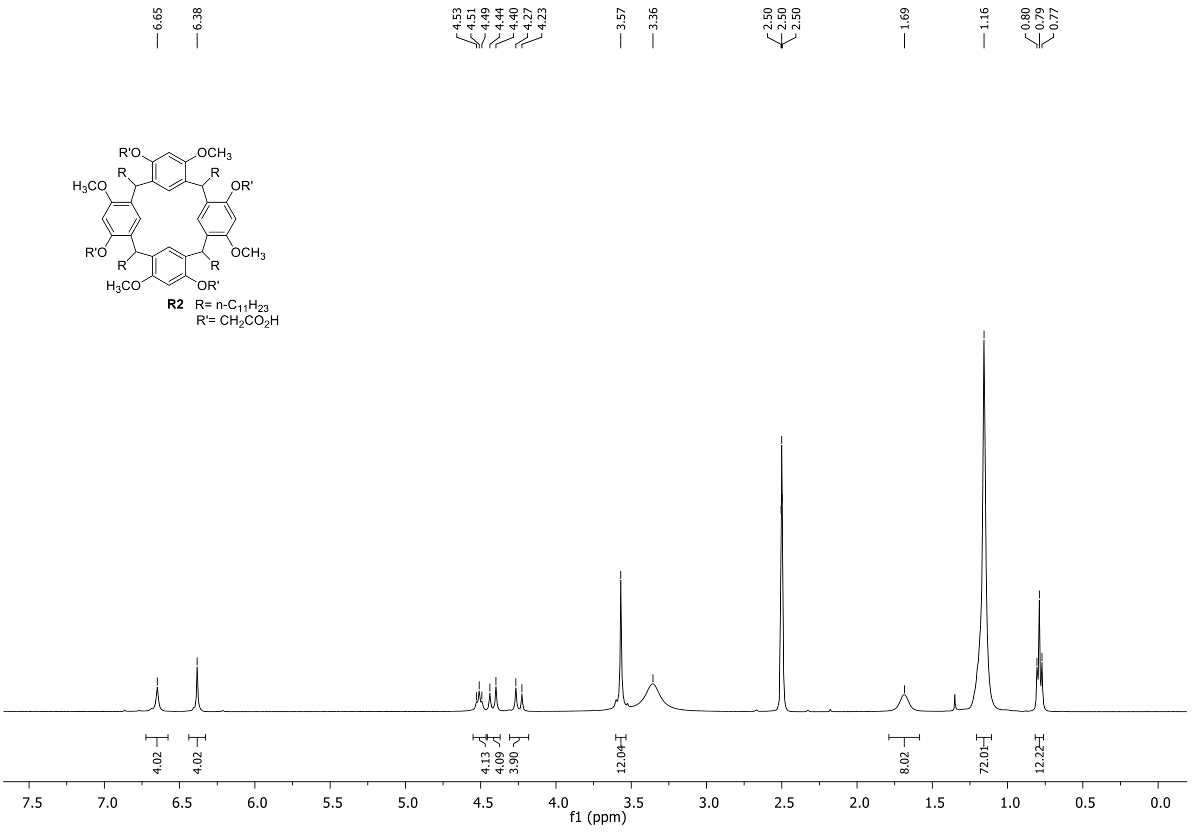

Fig. S6. ^{1}H NMR spectrum of compound **R2** (DMSO-d_6, 400 MHz).

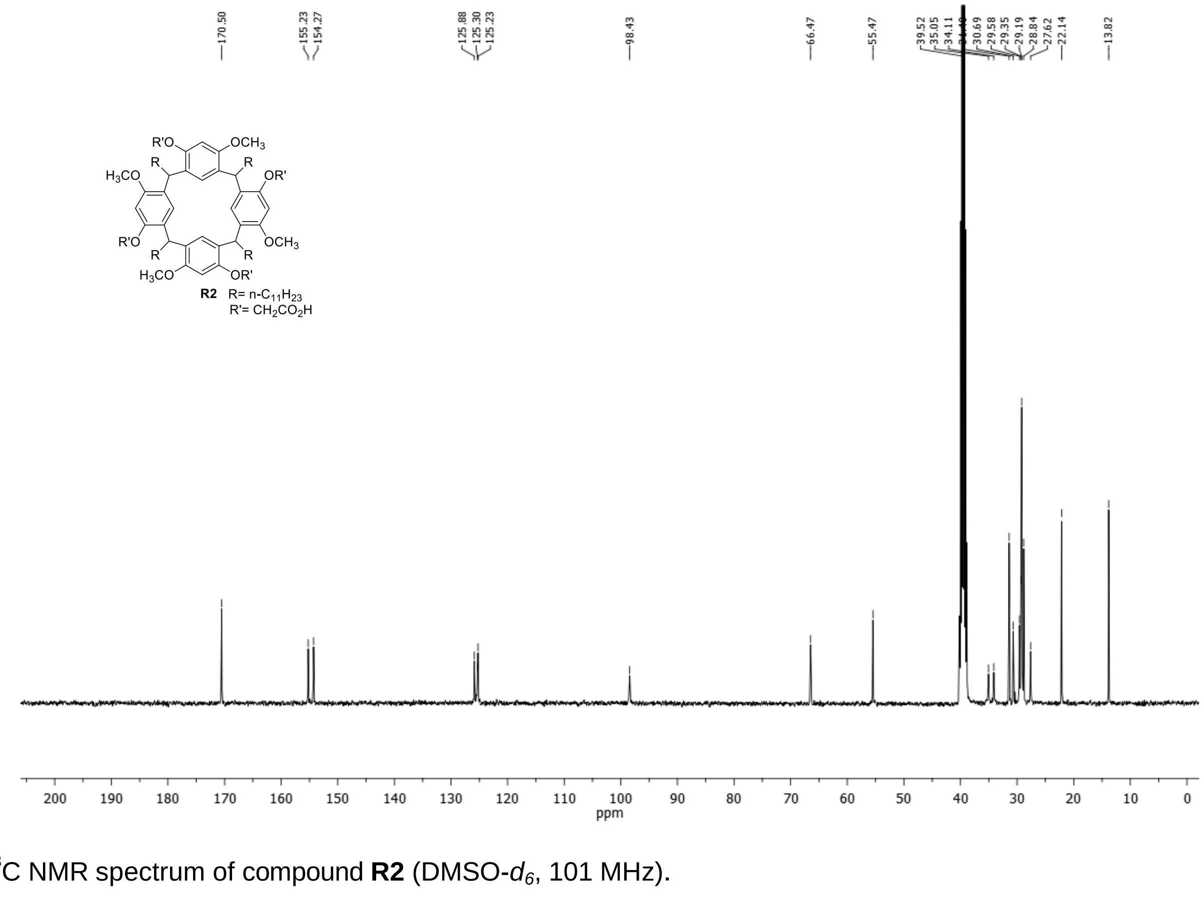

Fig. S7. ^{13}C NMR spectrum of compound **R2** (DMSO-d_6, 101 MHz).

3. ESI-HRMS of R2

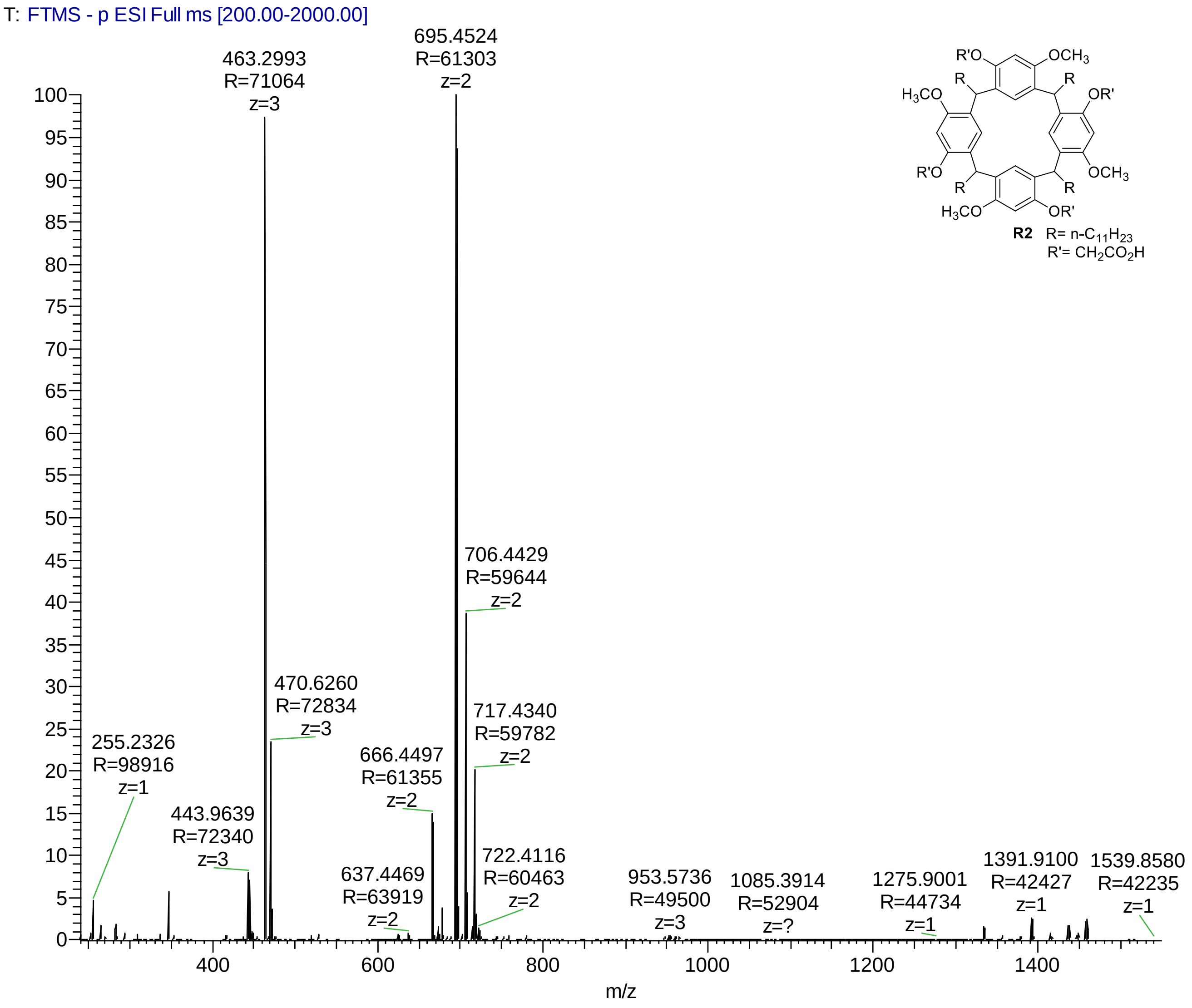

Fig. S8. ESI-HRMS spectrum of compound **R2**.

4. UV-visible experiments and DLS measurements

% H_2O	δ_H	$\Delta ABS_{(350-400)}$	Ø t = 0 h	Ø t = 1 h	var. %
68	18.82	0.01170	461.9	646.5	40.0
75	19.83	0.02750	702.5	1259.6	79.3
90	21.97	0.00311	259.6	298.8	15.1
95	22.69	0.00121	212.4	201.7	-5.0

Table S1. **R3** pH 9.96 from 68% to 95% H_2O.

h	Ø (nm)	var. %
0	276.4	0.0
1	275.1	-0.4
2	240.6	-12.9
4	135.7	-50.9
5	141.2	-48.9
21	315.8	14.3
24	363.6	31.6

Table S2. **R3** + **GlaB** pH 11.8, 95% H_2O.

	Sample in THF (mL)	H_2O (mL)	THF (mL)	Final water percentage	δ_H	Ø (nm) estimated values	$\Delta ABS_{(350-400)}$
1	0.3	0	1.700	0	9.1	222	0.00091
Baseline	0	0	2.000	-			
2	0.3	0.697	1.003	34.9	14.09	259	0.00286
Baseline	0	0.697	1.303	-			
3	0.3	1.020	0.680	51.0	16.39	273	0.00364
Baseline	0	1.020	0.980	-			
4	0.3	1.105	0.595	55.3	17.01	731	0.02821
Baseline	0	1.105	0.895	-			
5	0.3	1.156	0.544	57.8	17.37	2141	0.10390
Baseline	0	1.156	0.844	-			
6	0.3	1.190	0.510	59.5	17.61	2764	0.13731
Baseline	0	1.190	0.810	-			
7	0.3	1.360	0.340	68.0	18.82	2864	0.1427
Baseline	0	1.360	0.640	-			
8	0.3	1.700	0	85.0	21.26	3294	0.16579
Baseline	0	1.700	0.300	-			

Table S3. **R1**

	Sample in THF (mL)	H_2O (mL)	THF (mL)	Final water percentage	δ_H	Ø (nm) estimated values	ΔABS$_{(350-400)}$
1	0.3	0	1.700	0	9.10		0.001060
Baseline	0	0	2.000	-			
2	0.3	0.697	1.003	34.9	14.09		-0.000200
Baseline	0	0.697	1.303	-			
3	0.3	1.020	0.680	51.0	16.39		-0.000840
Baseline	0	1.020	0.980	-			
4	0.3	1.105	0.595	55.3	17.01		-0.000110
Baseline	0	1.105	0.895	-			
5	0.3	1.190	0.510	59.5	17.61		-0.000180
Baseline	0	1.190	0.810	-			
6	0.3	1.360	0.340	68.0	18.82	253.2	0.002570
Baseline	0	1.360	0.640	-			
7	0.3	1.500	0.200	75.0	19.83	839.0	0.034010
Baseline		1.500	0.500	-			
8	0.3	1.700	0	85.0	21.26	986.0	0.041900
Baseline	0	1.700	0.300	-			

Table S4. **R2** pH 6.21

	Sample in THF (mL)	H_2O/ buffer phosphate (mL)	THF (mL)	Final water percentage	δ_H	Ø (nm) estimated values	ΔABS$_{(350-400)}$
1	0.3	0	1.700	0	9.10		0.00138
Baseline	0	0	2.000	-			
2	0.3	0.697	1.003	34.9	14.09		0.00091
Baseline	0	0.697	1.303	-			
3	0.3	1.020	0.680	51.0	16.39	233.2	0.00150
Baseline	0	1.020	0.980	-			
4	0.3	1.105	0.595	55.3	17.01	289.3	0.00451
Baseline	0	1.105	0.895	-			
5	0.3	1.156	0.544	57.8	17.37	393.1	0.01008
Baseline	0	1.156	0.844	-			
6	0.3	1.190	0.510	59.5	17.61	1294.2	0.05844
Baseline	0	1.190	0.810	-			
7	0.3	1.360	0.340	68.0	18.82	3514.1	0.17758
Baseline	0	1.360	0.640	-			
8	0.3	1.500	0.200	75.0	19.83	4479.7	0.22940
Baseline	0	1.500	0.500	-			
9	0.3	1.700	0	85.0	21.26	6714.9	0.34936
Baseline	0	1.700	0.300	-			

Table S5. **R2** pH 1.91

	Sample in THF (mL)	H_2O/ buffer phosphate (mL)	THF (mL)	Final water percentage	δ_H	Ø (nm) estimated values	ΔABS$_{(350\text{-}400)}$
1	0.2	0	1.800	0	9.10		0.00127
Baseline	0	0	2.000	-			
2	0.2	0.897	0.903	44.85	15.51		-0.00187
Baseline	0	0.897	1.103	-			
3	0.2	1.600	0.200	80	20.54	249.1	0.00235
Baseline	0	1.600	0.400	-			
4	0.2	1.700	0.100	85	21.26	259.1	0.00289
Baseline	0	1.700	0.300	-			
5	0.1	1.900	0	95	22.69	370.0	0.00884
Baseline	0	1.900	0.100	-			

Table S6. **R2** pH 8.70

	Sample in THF (mL)	H_2O (mL)	THF (mL)	Final water percentage	δ_H	Ø (nm) estimated values	ΔABS$_{(350\text{-}400)}$
1	0.3	0	1.700	0	9.10		0.00079
Baseline	0	0	2.000	-			
2	0.3	0.697	1.003	34.9	14.09		0.00032
Baseline	0	0.697	1.303	-			
3	0.3	1.020	0.680	51.0	16.39		-0.000189
Baseline	0	1.020	0.980	-			
4	0.3	1.105	0.595	55.3	17.01	226.0	0.00111
Baseline	0	1.105	0.895	-			
5	0.3	1.190	0.510	59.5	17.61	650.4	0.02389
Baseline	0	1.190	0.810	-			
6	0.3	1.270	0.430	63.5	18.18	1229.0	0.05494
Baseline	0	1.270	0.730	-			
7	0.3	1.600	0.100	80.0	20.54	1087.5	0.04735
Baseline	0	1.600	0.400	-			
8	0.3	1.700	0	85.0	21.26	1025.9	0.04404
Baseline	0	1.700	0.300	-			
9	0.2	1.800	0	90.0	21.97	636.8	0.02316
Baseline	0	1.800	0.200	-			

Table S7. **R3** pH 6.21

	Sample in THF (mL)	H_2O/ buffer phosphate (mL)	THF (mL)	Final water percentage	δ_H	Ø (nm) estimated values	$\Delta ABS_{(350-400)}$
1	0.3	0	1.700	0	9.10		0.00079
Baseline	0	0	2.000	-			
2	0.3	1.020	0.680	51.0	16.39	223.2	0.00096
Baseline	0	1.020	0.980	-			
3	0.3	1.190	0.510	59.5	17.61	1279.5	0.05765
Baseline	0	1.190	0.810	-			
4	0.3	1.270	0.430	63.5	18.18	2890.7	0.14412
Baseline	0	1.270	0.730	-			
5	0.3	1.360	0.340	68.0	18.82	4010.9	0.20424
Baseline	0	1.360	0.640	-			
6	0.3	1.500	0.200	75.0	19.83	4776.3	0.24532
Baseline	0	1.500	0.500	-			
7	0.3	1.700	0	85.0	21.26	5460.0	0.28201
Baseline	0	1.700	0.300	-			

Table S8. **R3** pH 2.41

	Sample in THF (mL)	H_2O/ buffer phosphate (mL)	THF (mL)	Final water percentage	δ_H	Ø (nm) estimated values	$\Delta ABS_{(350-400)}$
1	0.3	0	1.700	0	9.1		0.00079
Baseline	0	0	2.000	-			
2	0.3	1.020	0.680	51.0	16.39	252.4	0.00253
Baseline	0	1.020	0.980	-			
3	0.3	1.190	0.510	59.5	17.61	980.6	0.04161
Baseline	0	1.190	0.810	-			
4	0.3	1.270	0.430	63.5	18.18	1610.4	0.07541
Baseline	0	1.270	0.730	-			
5	0.3	1.360	0.340	68.0	18.82	1093.3	0.04766
Baseline	0	1.360	0.640	-			
6	0.3	1.500	0.200	75.0	19.83	582.4	0.02024
Baseline	0	1.500	0.500	-			
7	0.3	1.700	0	85.0	21.26	310.4	0.00564
Baseline	0	1.700	0.300	-			

Table S9. **R3** pH 8.52

	Sample in THF (mL)	H_2O/ buffer phosphate (mL)	THF (mL)	Final water percentage	δ_H	Ø (nm) estimated values	$\Delta ABS_{(350-400)}$
1	0.3	0	1.700	0	9.1		0.00079
Baseline	0	0	2.000	-			
2	0.3	0.800	0.900	40	14.82	290.6	0.00458
Baseline	0	0.800	1.200	-			
3	0.3	0.900	0.800	45	15.54	344.1	0.00745
Baseline	0	0.900	1.100	-			
4	0.3	1.020	0.680	51.0	16.39	288.7	0.00448
Baseline	0	1.020	0.980	-			
5	0.3	1.190	0.510	59.5	17.61	232.7	0.00147
Baseline	0	1.190	0.810	-			
6	0.3	1.270	0.430	63.5	18.18	268.4	0.00339
Baseline	0	1.270	0.730	-			
7	0.3	1.360	0.340	68.0	18.82	423.3	0.0117
Baseline	0	1.360	0.640	-			
8	0.3	1.500	0.200	75.0	19.83	717.7	0.0275
Baseline	0	1.500	0.500	-			
9	0.3	1.700	0	85.0	21.26	325.3	0.00644
Baseline	0	1.700	0.300	-			
10	0.2	1.800	0	90.0	21.97	263.2	0.00311
Baseline	0	1.800	0.200	-			
11	0.1	1.900	0	95.0	22.69	227.8	0.00121
Baseline	0	1.900	0.100	-			

Table S10. **R3** pH 10.0

	Sample in THF (mL)	H_2O/ buffer phosphate (mL)	THF (mL)	Final water percentage	δ_H	Ø (nm) estimated values	$\Delta ABS_{(350-400)}$
1	0.3	0	1.700	0	0.0	9.10	220.0
Baseline	0	0	2.000	-			
2	0.3	0.900	0.800	45	15.54		-0.00072
Baseline	0	0.900	1.100	-			
3	0.3	1.020	0.680	51.0	16.393	336.6	0.00705
Baseline	0	1.020	0.980	-			
4	0.3	1.190	0.510	59.5	17.61	297.9	0.00497
Baseline	0	1.190	0.810	-			
5	0.3	1.270	0.430	63.5	18.18	275.0	0.00374
Baseline	0	1.270	0.730	-			
6	0.3	1.360	0.340	68.0	18.82	247.6	0.00227
Baseline	0	1.360	0.640	-			
7	0.3	1.500	0.200	75.0	19.83	262.1	0.00305
Baseline	0	1.500	0.500	-			
8	0.3	1.600	0.100	80.0	20.54	279.8	0.004
Baseline	0	1.600	0.400	-			
9	0.3	1.700	0	85.0	21.255	400.2	0.01046
Baseline	0	1.700	0.300	-			
10	0.2	1.800	0	90.0	21.97	253.5	0.00259
Baseline	0	1.800	0.200	-			
11	0.1	1.840	0.060	92.0	22.26	322.1	0.00627
Baseline	0	1.840	0.160	-			
12	0.1	1.900	0	95.0	22.69	320.0	0.00616
Baseline	0	1.900	0.100	-			
13	0	2.000	0	100	23.4	647.4	0.02373
Baseline	0	2.000	0	-			

Table S11. **R3** pH 11.8
